# Physical activity prescription for general practice patients with cardiovascular risk factors–the PEPPER randomised controlled trial protocol

**DOI:** 10.1186/s12889-019-7048-y

**Published:** 2019-06-03

**Authors:** David C. Missud, Elsa Parot-Schinkel, Laurent Connan, Bruno Vielle, Jean-François Huez

**Affiliations:** 1 Faculté de Médecine d’Angers, Angers, France; 2 Centre Hospitalier Universitaire d’Angers, Cellule Méthodologie et Biostatistiques, Angers, France

## Abstract

**Background:**

The health benefits of physical exercise have been shown to be important in the prevention of cardiovascular diseases in patients with hypertension, dyslipidaemia or diabetes. However, few strategies have demonstrated efficacy and practicality in the promotion of physical exercise among this group of patients in general practice.

**Methods:**

The PEPPER clinical study is a randomised controlled trial to evaluate the efficacy over a period of 12 months, in terms of physical activity level, of an intervention based on structured information delivery, a personalised written physical activity prescription in number of steps per day, a pedometer and a pedometer logbook, in 35 to 74-year-old patients with cardiovascular risk factors. 140 patients will be recruited in 15 GP practices and randomised in the intervention group or in the control group where patients will receive verbal advice of physical exercise. The primary outcome is the change at three months in total energy expenditure measured by an accelerometer over a 7-day period. Secondary outcomes include changes at 3 and 12 months in physical activity levels (accelerometer and International Physical Activity Questionnaire), quality of life (SF-36), blood pressure, weight, waist circumference, perceived obstacles to physical activity and patient compliance with the recommended strategy. Both groups will be compared using mixed models.

**Discussion:**

The results are expected at the end of 2019. If the intervention proves effective in durably increasing the level of physical activity, this strategy could be tested in a larger trial to examine its impact on cardiovascular diseases.

**Trial registration:**

US National Institutes of Health Clinical Trials Registry NCT02317003, December 15, 2014.

## Background

Insufficient physical activity represents the fourth highest mortality risk factor in the world with more than 3.2 million deaths annually and 32.1 million Quality Adjusted Life Year (QALY) [1]. All cause mortality risk is increased in sedentary individuals by 20 to 30% compared to individuals having moderate intensity physical activity for 30 min a day, four days a week [1]. The benefits of physical exercise are particularly significant for individuals with cardiovascular risk factors. A weekly 150 min physical activity of moderate intensity reduces the risk of developing hypercholesterolemia [2], the risk of coronary heart disease by 30%, the risk of diabetes by 27% [1], the risk of stroke by 25% [3] and of hypertension by 12% [4].

Recent cohort studies by Moore, et al. [5] and Wen, et al. [6], on large samples of hundreds of thousands of individuals in the USA, Sweden and China, have demonstrated significant benefits of physical exercise: 15 min of daily moderate intensity exercise produce a gain of 2.5 years of life compared to sedentary individuals, all cause mortality risk are reduced by 16% for active hypertensive or dyslipidaemic individuals compared to inactive individuals, and by 22% for the subgroup of diabetic or pre-diabetic patients. Both studies emphasize the high benefits of the first two hours of weekly physical exercise and have established a dose-response relationship of physical exercise: on top of 15 min of daily moderate intensity exercise, a daily additional 15 min yields a decrease of 4% of all cause mortality risk.

Various types of interventions have been evaluated over the past 15 years in order to promote physical activity and reduce sedentary lifestyle. Among the large variety of sometimes complex strategies that have been tested in the litterature, the most common, cost-effective, simple and consistently efficient components that were thought applicable to the context of this study were: patient education on the consequences of sedentary lifestyle and the benefits of physical exercise [7], a written prescription of physical exercise with measurable goals [8], encouraging early identification of barriers to behavioural change [9], self-determined objectives [10], setting graded tasks [7, 11], self-monitoring of behaviour and performance [7, 11], the use of a pedometer [12–15] and using follow-up prompts and encouragement through meetings, phone calls or messages [11]. However, in many countries, the most prevalent strategy to promote physical activity in primary care remains verbal advice to patients [16], with limited results.

Moreover, few studies have evaluated the long-term motivational effect of pedometers (beyond 6 months). As stated by the British National Institute for Clinical Excellence [17], although pedometers have shown short term efficacy, there is currently insufficient evidence to generalise their use in primary care. In addition, the most efficacious interventions are often complex, involving regular follow-up phone calls or meetings with the support of nurses or other health professionals. These strategies are, therefore, often impractical in countries like France, where general practitioners have limited support.

The aim of the present study is, therefore, to test a simple, low-cost, primary care intervention to increase the physical activity of patients with cardiovascular risk factors.

## Methods/design

### Study design

The PEPPER study is an experimental, comparative, prospective, open, multicentric controlled randomised trial in two parallel groups.

### Participants

Participants are recruited by their general practitioner (GP) in 15 GP practices in the Pays-De-La-Loire region.

Participants are 35 to 74-year-old adults, consulting their usual general practitioner (GP) for a non-urgent matter, and followed-up every three months for hypertension, hypercholesterolemia or non-insulin dependent type 2 diabetes. Patients will be judged insufficiently active by their general practitioner on the basis of at least their answers to two questions:

- “Do you practice any sports, including cycling, an hour or more every week?”
- “Does your work involve physical activities?”

The inclusion criterion is fulfilled by a negative answer to both questions.

Exclusion criteria are: having a health condition that contraindicates a moderate physical activity, as judged by the general practitioner; being unable to walk without human or material help; having any health condition, including psychiatric or cognitive impairments, that would prevent the participant from fully understanding the study and his or her doctor’s advice, as judged by the general practitioner; being unable to communicate in French, or refusing to participate.

Patients will be able to withdraw their consent or request to leave the study at any time and regardless of the reason.

The investigator can discontinue, temporarily or permanently, the participation of a patient in the study for any reason that would be in the best interest of the patient, especially in case of serious adverse events.

The exclusion of a patient is required in case of:

- Withdrawal of consent for participation,
- Serious event with consequences for the patient’s ability to follow the study protocol.

### Control group (VA)

In the control group, participants who are judged insufficiently active by their GP will be verbally advised to increase their physical exercise to at least 15 min of brisk walking, or other physical activity that makes them breathe quicker than normal, every day of the week.

Doctors will deliver the intervention every three months at each follow-up consultation.

### Intervention group (PPIL)

The strategy in the intervention group involves:

- A written prescription of physical activity,
- Information on the benefits of physical activity,
- The loan of a pedometer,
- The delivery of a pedometer logbook.

#### Prescription of physical exercise

The prescription of physical exercise written by the GP states the number of steps per day above the patient’s baseline level. For example, the GP may prescribe “4000 steps above your usual number of steps”. The GP is free to adjust the prescription. The patient should agree with the prescription and should regard the objective as being achievable. The GP will encourage the patient to try and reach the prescribed goal and to define intermediate self-determined weekly goals in order to achieve the final objective in about three months. The patient and the GP will reassess the objective every three months during follow-up consultations.

#### Information on physical activity

The GP will deliver verbal and written information on the benefits of physical activity, included in the pedometer logbook given to the patient. The information is structured according to behavioural and cognitive theories of behaviour change, and refers to the following strategies as coded by Abraham and Michie [18]:

Provide information on the consequences of behaviour in general, emphasising short term and long term effects,
Provide information on the consequences of behaviour to the individual, in relation to the patient’s specific risk factors or pathology,
5. Goal setting,
8. Barrier identification/Problem solving, using the Determinant of Physical Activity Questionnaire [9] included in the logbook,
9. Set graded tasks, through weekly self-determined objectives,
10. Prompt review of behavioural goals, during follow-up consultations and in review of previous performances in the logbook,
16. Prompt self-monitoring of behaviour, using the pedometer,
19. Provide feedback on performance, during follow-up consultations.

#### Pedometer

The GP will lend a pedometer (Omron HJ-321-E) to the patient. The pedometer is tri-axial to make it easy to carry in a pocket or a bag. It has a 7-day memory and resets automatically at midnight. The GP will advise the patient to carry the pedometer as often as possible, and to regularly monitor the number of steps achieved during the day.

#### Logbook

The logbook will begin with information on the benefits of physical exercise, a simplified pedometer user manual, suggestions on how to increase the number of daily steps and a frequently asked questions section. The logbook itself will include a weekly diary where the patient records his or her self-determined weekly objective and the number of daily steps achieved, and the average number of steps achieved during the past seven days. For activities that are not well accounted for by the pedometer (swimming, cycling, lifting heavy weights…), the end of the logbook will include a table giving the equivalence in number of steps for various physical activities. The GP will encourage the patient to bring their logbook to follow-up consultations to review his or her progress.

The GP will administer the intervention at least every three months during each follow-up consultation.

### Recruitment and follow-up

GP practices are selected according to the following criteria:

- The GP practice is located in the pays De La Loire region in France
- The general practitioner is a GP trainer involved in the University of Angers Medical School
- The general practitioner volunteers to be investigator in this study

All the investigators are briefed on the details of the study and the delivery of the interventions.

The GPs will recruit participants from among their patients who meet the inclusion criteria, and agree to participate in the study, during normal consultations. The inclusion period is set to 15 weeks and may be prolonged according to the number of participants actually recruited.

Each participant will receive by mail, after his or her inclusion, a self-assessment kit, including the accelerometer to wear for at least seven days in a row, from sunrise to sunset, and self-administered questionnaires. After the 7-day period, participants will return the self-assessment kit by mail.

Participants will then meet again with their GPs. During this second consultation, participants will be randomised to either the PPIL or the VA group and GPs will deliver the assigned intervention.

**Fig. 1:**
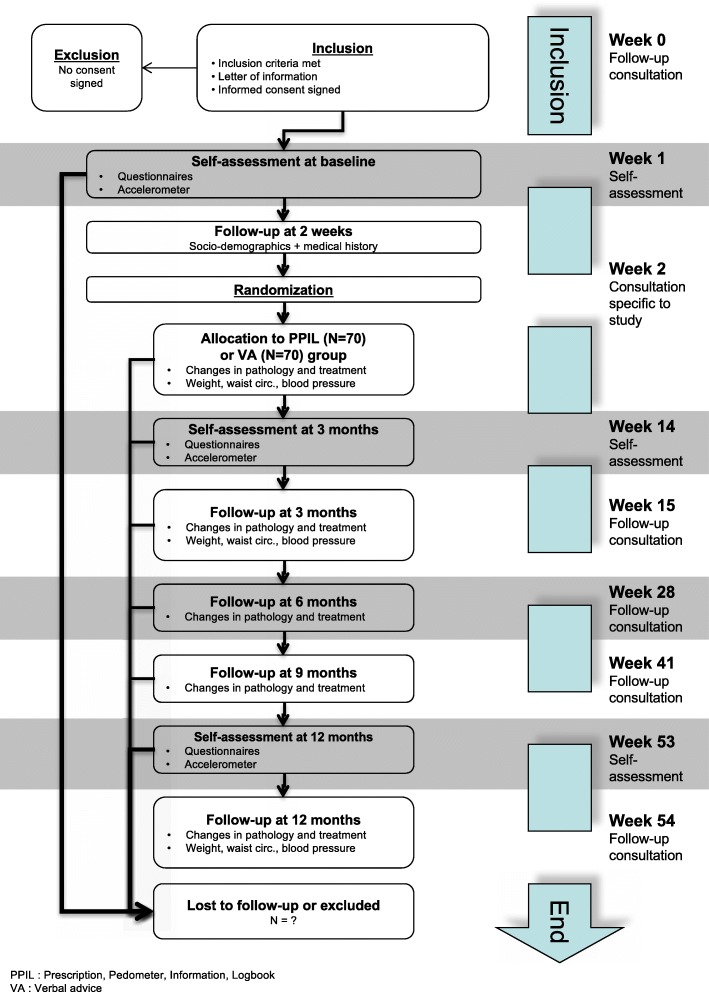
Recruitment and follow-up flowchart

Follow-up consultations will then occur every three months during the following 12 months. Follow-up consultations at 3 and 12 months will be preceded by a 7-day self-assessment period using the accelerometer and the questionnaires. The recruitment and follow-up flowchart is given in Fig. 1.

### Hypothesis and objectives

The study hypothesis is that “Prescription, Pedometer, Information, Logbook” intervention (PPIL) is better from Verbal Advice (VA) in increasing the physical activity of patients with cardiovascular risk factors.

The primary objective of the study is to determine whether PPIL is better from VA in increasing the weekly energy expenditure of participants at three months.

The key secondary objective is to determine whether PPIL is better from VA in increasing the weekly energy expenditure of participants at 12 months.

The other secondary objectives are to determine whether PPIL is better from VA in improving quality of life, reducing waist circumference, weight and arterial blood pressure, and to describe the deviations of doctors and patients from the recommended strategies.

### Primary and secondary outcomes

The primary outcome is the change at three months of the total weekly energy expenditure, measured by an accelerometer in Metabolic Equivalent Task-minute (MET-min), compared to baseline. The accelerometer (Actigraph wGT3x-BT validated in research [19–23]) is worn at the belt over a 7-day period, from morning to bedtime. The Swartz Adult Overground & Lifestyle algorithm [24] is used to convert from accelerometer counts to MET.

The key secondary outcome is the change at 12 months of the total weekly energy expenditure, measured by an accelerometer in MET-min, compared to baseline.

The other secondary outcomes are:

- Change at 3 and 12 months in the weekly time dedicated to moderate and high intensity physical activity, and in the weekly physical activity level in MET-min calculated by the International Physical Activity Questionnaire (IPAQ), from baseline.
- Change at 3 and 12 months in the scores on the SF36 quality of life questionnaire compared to baseline.
- Change at 3 and 12 months to weight, waist circumference and arterial blood pressure compared to baseline,
- Changes in the patient’s drug treatment at 3 and 12 months,
- Perceived obstacles to physical activity at 3 and 12 months as assessed by the Determinants of Physical Activity Questionnaire (DPAQ),
- Details of the physical exercise promotion strategies applied during the study,
- Compliance of patients with their doctor’s recommendations.

### Sample size

Studies by Kirk, et al. [25], Pinto, et al. [26], Baker, et al. [27] and Mutrie, et al. [28] were chosen as a base for sample sizing, considering their similarity to the PEPPER trial, their sample size and the outcomes reported. None of these studies reported measures in MET-min, but there is a linear relationship between the energy expenditure and the duration of physical activity, the step count or the raw accelerometer output: 15 min of moderate intensity physical exercise is equivalent to 45 MET-min.

Using a 2-tailed hypothesis, a sample of 140 patients in two groups of 70 will be necessary to detect a difference, between the two groups, of the change in total energy expenditure at three months of 105 MET-min/week (about 35 min of moderate intensity physical exercise per week), with an estimated standard deviation of 185 MET-min/week, an alpha risk of 5% and a power of 90%.

### Randomisation and blinding

Randomisation will occur at the first follow-up consultation after the first self-assessment period. Participants will be randomised to either the PPIL or the VA group by their GP. The group allocation will be achieved by a computerised dynamic randomisation using a minimisation algorithm based on nine criteria: diabetes (yes/no), body mass index (BMI) (< 30, ≥30), sex (female/male), hypertension (yes/no), employment status (employed/unemployed), hypercholesterolemia (yes/no), age (35-54y, 55-74y), education level (≤ A-level, > A-level) and follow-up doctor. The randomisation procedure will be concealed, centralised and performed by the Cellule de Méthodologie et Biostatistiques of Angers University Hospital.

The randomisation system will be internet-based with investigator access 24 h a day, seven days a week using a personal ID and PIN code.

Considering the nature of the interventions, it is not possible to blind investigators or patients.

### Data collection

Data from the study are collected during follow-up consultations and by self-assessment (questionnaires and accelerometer), with telephone reminders to patients and physicians in case the data are not received.

#### Socio-demographic data

The following data are collected at the beginning of the study: age, sex, education level, family unit (alone / with family), socio-professional category (1 - farmer, 2 -craftsman/trader/small employer, 3 - higher managerial and professional occupations, 4 - intermediate occupations, 5 - lower supervisory occupations, 6 - lower technical occupations, 7 - retired, 8 - other inactive), occupation rate (> 80%, ≤80%), employment type (physical / lightly physical / sedentary), living environment (urban, semi-urban, rural) and distance to workplace (≤5 km, between 5 and 10 km, > 10 km).

#### Risk factors and medical history

At inclusion, GPs will gather the following data: tobacco use, hypercholesterolemia, arterial hypertension, diabetes, significant medical history (cardiovascular, respiratory, orthopaedic, neurological diseases…) and drug history.

#### Energy expenditure and physical activity

Energy expenditure and physical activity will be measured at 0, 3 and 12 months, by two methods:

- Using an accelerometer (Actigraph wGT3x-BT), worn at the waist using an elastic belt during seven consecutive days, from morning to bedtime, to measure the total weekly energy expenditure in MET-min/week, the total time spent in moderate (between 3.75 and 7.49 MET-min) and high intensity (> 7.5 MET-min) physical activities; The default energy expenditure during the time when the accelerometer is not worn will be 1 MET-min.

- Using the self-administered International Physical Activity Questionnaire (IPAQ) [29], short version, covering the past seven days, to measure the weekly energy expenditure in MET-min/week.

#### Quality of life

Quality of life will be measured at 0, 3 and 12 months using the eight scores of the Short Form 36 (SF-36) self-administered questionnaire [30].

#### Clinical and morphological measures

GPs will assess the following data at 0, 3 and 12 months: systolic and diastolic arterial blood pressure, weight, height, waist circumference (National Institutes of Health method [31, 32]).

#### Change in health status and adverse events

Data are collected at 0, 3 and 12 months regarding changes in drug treatment (stable / increased / decreased), adverse events (acute serious pathological episode, new diagnostics of chronic or serious pathologies…) and, if available, the dates and values of the latest blood tests, including total cholesterol, HDL-C, LDL-C, triglycerides and HbA1c.

#### Perceived obstacles to change

Perceived obstacles to change will be assessed at 3 and 12 months using the self-administered Determinants of Physical Activity Questionnaire (DPAQ) [9].

#### Monitoring of patient compliance with and deviations from the protocol

The data collection plan includes the description of any deviation from the protocol in terms of physical exercise promotion strategy and the compliance of participants with their GP’s recommendations, in both the intervention and the control group. In particular, the following data will be collected in order to evaluate the study process:

- Compliance of GPs with the intervention (structured information given to patient, logbook given to patient, pedometer given to patient, content of the physical activity prescription…)
- Compliance of patients with the process: attendance to consultations, use of accelerometer, use of pedometer, percentage of logbook data filled...

### Data management and quality control

All data will be collected using an electronic Case Report Form (CRF), with all data stored centrally in Angers University Hospital. The data management team will be in charge of carrying out quality controls and monitoring of data modifications.

### Statistical analyses

A diagram will describe the participant selection process, with a descriptive analysis of their characteristics.

Quantitative variables will be expressed as mean ± standard deviation (median and inter-quartile interval if the normality hypothesis is not valid). Qualitative variables will be expressed as frequency and percentage.

Intention to treat analysis will be carried out, taking into account all randomised people. Multiple imputation techniques will be used to handle missing data.

An additional per-protocol analysis will also be carried out, taking into account those patients for whom the assessment criteria will be available.

The Student’s t-test (or the non-parametric Mann-Whitney U-test, depending on the variable distribution) and mixed models will be used to compare both groups for the primary outcome and for the other quantitative variables.

For qualitative variables, the Pearson’s chi-squared test (or the Fisher’s exact test, when necessary) will be used.

The level of significance is set to 0.05 and all tests are bilateral.

Bivariate analyses will be carried out for all outcomes. Additional multivariate analyses may be performed to adjust for significant factors. Backward stepwise regression techniques will be used to select confounders. Adjustment variables will include:

Sex (F/M),

Age (35-54y, 55-74y),

Education level (≤ A-level, > A-level),

BMI (< 30, ≥ 30),

Diabetes (yes/no),

Occupational rate (> 80%, ≤80%).

Living environment (urban, semi-urban, rural).

All hypotheses and tests are two-tailed.

## Discussion

Results are expected at the beginning of 2019. We expect the PPIL intervention to be better than the VA intervention. If the results meet our expectations, the PPIL intervention, being simple, and not requiring important staff and financial resources, could easily be promoted towards GPs on a large scale to improve the physical activity of a notable number of patients.

### Strengths and limitations of this study

- This is a multi-centre, open, randomised controlled study design. Considering that the interventions are different in nature, there is no possible blinding in the intervention or in the collection of anthropometric data.
- We considered it was impractical and too difficult to ensure to ensure that randomized patients do not meet each other or share information about the study. Therefore there is a possibility that some patients included in the study and assigned to different groups knew each other and were able to share details about the study. It is also possible that some patients included in the control group (verbal advice) decided on their own to use a pedometer that influenced their physical activity.
- The physical activity of patients is assessed objectively (primary outcome) using accelerometers and questionnaires instead of questionnaires alone. However there is a possibility that the fact of wearing the accelerometer had an impact on the physical activity of the patient. It is also possible that some patient did not wear the accelerometer appropriately because they found it cumbersome.
- The intervention studied was designed to ensure minimal deviation from usual patient follow-up, in order to make the intervention easier to apply, to limit the workload of GPs, and to limit the financial cost of additional consultations.
- The simple intervention requires limited resources, including limited staff, financial input and time, and limited changes to usual practice.
- Considering the small recruitment size, the intervention effect on health outcomes, such as control of hypertension, control of glycaemia, or improvement in dyslipidaemia, is beyond the study statistical power. Therefore the primary outcomes of the study are only surrogate outcomes. However the available literature gives us confidence that increased physical activity as measured in this study will probably lead to improved health outcomes.
- Considering the risk of withdrawal at 1 year and the relatively small sample size, primary outcomes are considered at 3 months rather than at 1 year.

## Data Availability

No later than two years after the end of the trial, de-identified data will be available on request for sharing purposes by contacting Pr. Laurent Connan (laurent.connan@univ-angers.fr). All trial documents and statistical codes for generating the results will also be shared. The steering committee will approve all study reports before submission for publication. The study results will be released to the participating general practitioners, patients and the general medical community.

## Abbreviations

BMI: Body Mass Index
CRF: Case Report Form
DPAQ: Determinants of Physical Activity Questionnaire
GP: General Practitioner
IPAQ: International Physical Activity Questionnaire
MET: Metabolic Equivalent Task
MET-min: Metabolic Equivalent Task-minute
PPIL: Prescription, Pedometer, Information, Logbook
QALY: Quality Adjusted Life Years
SF36: Short Form 36
VA: Verbal Advice
